# Livor Mortis and Forensic Dermatology: A Review of Death-Related Gravity-Dependent Lividity and Postmortem Hypostasis

**DOI:** 10.7759/cureus.90760

**Published:** 2025-08-22

**Authors:** Philip R Cohen, Ronald J Moss, Joseph A Prahlow

**Affiliations:** 1 Dermatology, University of California, Davis Medical Center, Sacramento, USA; 2 Dermatology, Touro University California College of Osteopathic Medicine, Vallejo, USA; 3 Maples Center for Forensic Medicine, University of Florida College of Medicine, Gainesville, USA; 4 Pathology, St. Louis University School of Medicine, St. Louis, USA; 5 Forensic Pathology, Office of the Medical Examiner, St. Louis, USA

**Keywords:** color, cutaneous, dermatology, fatal, forensic, hypostasis, lividity, livor, mortis, postmortem

## Abstract

Livor mortis is an early postmortem change; it is also referred to as dependent lividity or postmortem hypostasis. This is a narrative review; the PubMed search engine was used to obtain citations of relevant articles. The following terms, by themselves and in combination, were used to screen for appropriate published manuscripts that described the features of livor mortis: color, cutaneous, dermatology, fatal, forensic, hypostasis, lividity, livor, mortis, and postmortem. The articles obtained from those searches were reviewed, and the relevant references cited by the articles were evaluated. Lividity typically presents as small blanchable red-purple macules and patches on the dependent areas of the decedent; it is usually observed within two hours after the person has died; however, it can be noted as early as 20 minutes after death. After four to six hours, hypostasis becomes more readily apparent; the smaller lesions become confluent and occur in larger regions of the body. However, contact pallor is observed in the dependent areas affected by direct pressure; these sites do not become red-purple. Livor mortis is usually “fixed,” and not blanchable, after eight to 12 hours. A bruise is the most common condition in the clinical differential diagnosis of lividity. The onset and duration until fixation of postmortem hypostasis are variable; lividity is also influenced not only by temperature but also by other factors. Livor mortis has at least three potential applications in forensic medicine. The first is that lividity, usually in combination with information from other postmortem changes, can be used to estimate the time since death; however, livor mortis is not reliable as an independent observation for establishing a range for the postmortem interval. The second is that livor mortis, when fixed, can be very helpful to establish that a decedent has been moved after death. The third is that the color of the lividity may possibly provide information regarding the cause of death; for example, carbon monoxide intoxication, hypothermia, or cyanide poisoning can be associated with cherry-red or pink lividity. New discoveries in medical technology have provided the ability to incorporate the use of spectrophotometric analysis of lividity to provide an objective assessment of postmortem hypostasis and correlate the information to more accurately estimate the postmortem interval.

## Introduction and background

Introduction

Livor mortis is an acquired discoloration of the skin of a decedent that begins shortly after the death of the individual. It is also referred to as lividity or hypostasis. Adjectives are occasionally included such as dependent lividity or postmortem hypostasis [1-14].

Background

Livor mortis is an early postmortem change; it begins as soon as the heart stops beating. It is a gravity-dependent process. The affected skin usually presents with a blanchable red-purple color. Subsequently, the lividity can no longer be blanched when pressure is applied, and it can eventually become more purple in appearance [1-14]. Temperature and other environmental factors, such as exposure to air, movement of the body, and clothing, can affect the appearance of lividity.

Livor mortis may be helpful as a method to estimate the postmortem interval. Lividity may be useful when the time since death ranges between about two hours (when lividity is typically perceptible) and approximately eight hours (when lividity is fixed). However, the time required for postmortem hypostasis to become fixed varies greatly from person to person and has been observed from six to 12 hours [1-13].

It is important to collaborate the observations made with regard to livor mortis with those of other methods that assess postmortem changes to establish a valid and reliable estimation of the time since death [15]. Livor mortis, when fixed, can be useful for establishing the terminal position of the body when the lividity becomes fixed and whether the body has been moved after this time. In addition, in certain circumstances, livor mortis may provide salient clues to the cause of death [1-14].

Key contributions to the history of livor mortis are summarized and illustrative case investigations that have been influenced by the information provided by postmortem hypostasis are described. The clinical differential diagnosis of livor mortis is presented and factors that can result in variability in the onset or progression of lividity are listed. The utility of livor mortis in forensic investigations are discussed; these include not only livor mortis contributing to establishment an estimation of the range of the postmortem interval and providing possible information regarding the position of the decedent at the time of death and whether the body has been repositioned or moved, but also the potential of the livor mortis to elucidate critical insight into the cause of death based on lividity color. And finally, new and innovative evaluation of livor mortis using spectrophotometry to evaluate lividity and the application of the results to more accurately provide an estimation of the time since death are presented.

Search strategy

This is a narrative review; the PubMed search engine was used to obtain citations of relevant articles. The following terms, by themselves and in combination, were used to screen for appropriate published manuscripts that described the features of livor mortis: color, cutaneous, dermatology, fatal, forensic, hypostasis, lividity, livor, mortis, and postmortem. The articles obtained from those searches were reviewed and the relevant references cited by the articles were evaluated.

## Review

History and landmark contributions

The description of livor mortis was initially published in the first forensic handbook in 1247 [16]. Lividity also appeared in subsequent textbooks in the mid-1800s [17]. In addition, landmark contributions of livor mortis playing a role more recently in establishing the cause of death during criminal investigations are described [18-21].

The Washing Away of Wrongs

The Washing Away of Wrongs is acknowledged to be the oldest textbook in forensic science. It appeared in 1247 during the Song Dynasty in China. It is also known as “Xi Lu,” “Hsi Duan Yu,” and “Collected Cases of Injustice Rectified” [16].

The book had been written by Song Ci (1186-1249 AD). He was a physician; he was also known as the father of Chinese medicine. In addition, he was a senior criminal court judge [16].

When Song Ci described livor mortis, “he noted how when the blood inside of the belly disperses on the outside. (He observed that) it cannot amass, so (it) floats (around the rest of the body) [16].” In his writings, he characterized the difference between bruising and livor mortis. He also described how the patterns of lividity could reveal whether the body had been moved after death [16].

Principles of Forensic Medicine and Principles of Medical Jurisprudence

The Principles of Forensic Medicine was written by Dr. William Augustus Guy in 1844; the following year, the first American edition was published. Dr. Guy was a physician and had been appointed as a Professor of Forensic Medicine at King’s College in London from 1843 to 1868; he was also the Dean of the Faculty of Medicine at the College from 1846 to 1858. In addition, he was a prominent statistician and was the Honorary Secretary of the Statistical Society from 1843 to 1868 [17].

The Principles of Medical Jurisprudence was written by Amos Dean, a Professor of Medical Jurisprudence in the Albany Medical College, in 1850. He played a major role in founding the Albany Law School. Until his death in 1868, he was the principal manager of the Law School [17].

Professor Dean repeated Dr. Guy’s writing in his book. Dr. Guy had described the principal signs of death; there were 10. One of the signs regarded the presentation of the skin. The comments regarded not only the presence of pallor and lividity, but also the loss of elasticity [17].

A Handbook of the Practice of Forensic Medicine Based Upon Personal Experience

Johann Ludwig Casper (1796-1864) was a German author, criminologist, forensic scientist, pathologist, pediatrician, and pharmacologist. He was also a professor at both the Medicinal College of the Province of Brandenburg (1822) and the Medicina Forensis and Publica (1839); subsequently, he became the director of a forensic medicine institute (1841). He is best known for his ratio that defines the time required for a body to putrefy; Casper’s Dictum states that putrefaction of a body varies depending on what substance the body is in; the ratio is 1:2:8 depending on whether the body is in air, water, or earth, respectively [17].

Dr. Casper’s translation of the German book on Forensic Medicine was published in 1864. The book describes the sequence of events that occur from death to putrefaction and specifically comments on livor mortis. Dr. Casper stated “hypostasis results from gravitation of the blood in the capillaries in obedience of the laws of inert matter. They form between eight and 12 hours and increase in size until the commencement of putrefaction. They are themselves sufficient evidence of the reality of death [17].” Dr. Casper also commented regarding how inexperienced individuals would confuse hypostasis with ecchymoses [17].

Criminal Investigation

Evaluation of livor mortis has been used to determine if the decedent has been moved since dying; the pattern of lividity has been crucial in the investigation of death and in some circumstances establishing that the cause of death was homicide. Two examples in which livor mortis had a substantial role in the criminal investigation include the murder of Hae Min Lee and the death of Evangelina Wing.

Murder of Hae Min Lee: The murder of Hae Min Lee in January 1999 by Adnan Masud Syed is a case that had captured national attention in the United States of America. It had been featured in the podcast Serial; subsequently, it was also the subject of another podcast, Undisclosed: The State vs. Adnan Syed. Two books were published about the case and a four-hour television documentary was produced. With regard to forensic medicine, the pattern of livor mortis observed on the decedent did not correlate with the presentation of the evidence by the prosecution [18,19].

Hae Min Lee was an 18-year-old high school student who went missing on January 13, 1999; she had been 5 feet 6 inches tall and had weighed 134 pounds. On February 9, 1999, her corpse was discovered in Leakin Park, Baltimore, Maryland; her body was found on its right side in the ground. Lee’s ex-boyfriend Adnan Masud Syed (17 years old) was subsequently sentenced to life in prison plus 30 years for charges of kidnapping, false imprisonment, robbery, and first-degree murder [18,19].

When Lee’s body was found, livor mortis was present and fixed on the anterior surface of the body, except in areas exposed to pressure. Rigor mortis was broken to an equal degree in all extremities. The autopsy revealed that the cause of death was strangulation [18,19].

The prosecuting attorney proposed that Lee had been placed in the trunk of her 1998 Nissan Sentra for about five hours and then buried in Leakin Park. The State’s key witness commented that Lee was pretzeled up in the trunk (presumably on her side). The witness also testified that she had been buried on her right side [18,19].

Importantly, Lee’s body had fixed frontal lividity. The postmortem hypostasis was evenly present on the front of her torso; dependent lividity was not present on the right side of Lee’s body. Therefore, for at least several hours (from at least five to perhaps more than eight to 12 hours), Lee had to have been lying in a prone position (on her abdomen with her face down) before being transported to the burial site in Leakin Park. In conclusion, based on the distribution of livor mortis, Lee’s body had been moved many hours after her death [18,19], or more specifically, many hours after the lividity had become fixed.

During the years after sentencing, there were several appeals for a retrial. Finally, on March 6, 2025, a judge reduced Syed’s sentence to time served [19]. It has been suggested that “the livor mortis evidence in the Lee case might [have] actually play[ed] a role in reversing the trial outcome and life sentence of her convicted killer, Adnan Syed [18].”

Death of Evangelina Wing: On December 20, 2014, emergency responders arrived at the home of Dorothy Wing (age 24 years old) in Seaside, Oregon. Wing lived there with her boyfriend Randy Roden (26 years old). They found her two-year-old daughter, Evangelina Wing dead. They also found that her two-year-old brother (who was 11 months older) and five-year-old brother both had injuries [18,20,21].

Evangelina was lying on her side; she was on a bed in her mother and Roden’s room. She was in full rigor mortis. However, her entire back showed fixed livor mortis [18,20,21].

Importantly, based on the location of Evangelina’s lividity, the distribution was not consistent with the position that she was found by the first responders. It was apparent that Evangelina had been moved since the time of death and that Evangelina had been dead for several hours; based on the postmortem hypostasis and presence of full rigor mortis, the time since death could have been between eight and more than 12 hours [18,20,21].

The remainder of her gross examination showed Evangelina had burns on her limbs, an untreated broken left arm, and periorbital ecchymoses. Evangelina had alleged bite marks on her arms. An autopsy determined that Evangelina died of blunt force trauma [18,20,21].

Dorothy Wing was sentenced in 2016 to 15 years in prison for first-degree manslaughter and two counts of first-degree criminal mistreatment. Randy Roden was sentenced in 2016 to 35 years in prison for murder by abuse, felony murder, manslaughter, criminal mistreatment and assault related to the abuse of Dorothy Wing’s other two children. However, in 2019, the Oregon Court of Appeals overturned the murder conviction since the alleged bite marks were wounds from methicillin-resistant Staphylococcus aureus infection; to avoid a retrial, Randy Roden settled on a manslaughter guilty plea and a sentence of 20 years from his 2014 arrest [18,20,21].

Clinical presentation

Onset of Livor Mortis

The onset of livor mortis begins immediately after death. Once the heart stops pumping, circulation of blood ceases. Thereafter, because of gravity, the blood in the body begins to move toward the dependent areas [1-14].

Postmortem hypostasis may be observed as early as 20 minutes after death [1,2]. It begins as focal patches that can be noted in the dependent areas of the body. Within two hours after death, livor mortis has typically become clinically apparent (Table 1) [1-13].

**Table 1:** Temporal appearance of livor mortis

Initially visible (hours)	Readily observed (hours)	Fixed (hours)	Reference
0.33	Not stated	8-12	[1]
0.33-0.5	Not stated	Greater than 12	[2]
0.5-2	6-12	Greater than 12	[3]
0.5-2	Not stated	6-12	[4]
0.5-2	Not stated	6-12	[5]
0.5-2	Not stated	8-12	[6]
0.5-2	Not stated	8-12	[7]
1	2-4	8-12	[8]
1	3-4	6-8	[9]
1-3	4-6	6-8	[10]
Not stated	Not stated	8	[11]
Not stated	Not stated	8-12	[12]
3-4	6-8	10-12	[13]

Continued Development of Livor Mortis

The red-purple patches of lividity gradually increase in size. They also spread throughout the gravity-dependent regions of the body [10]. Between two and six hours, livor mortis is usually well established [1-14].

**Figure 1:**
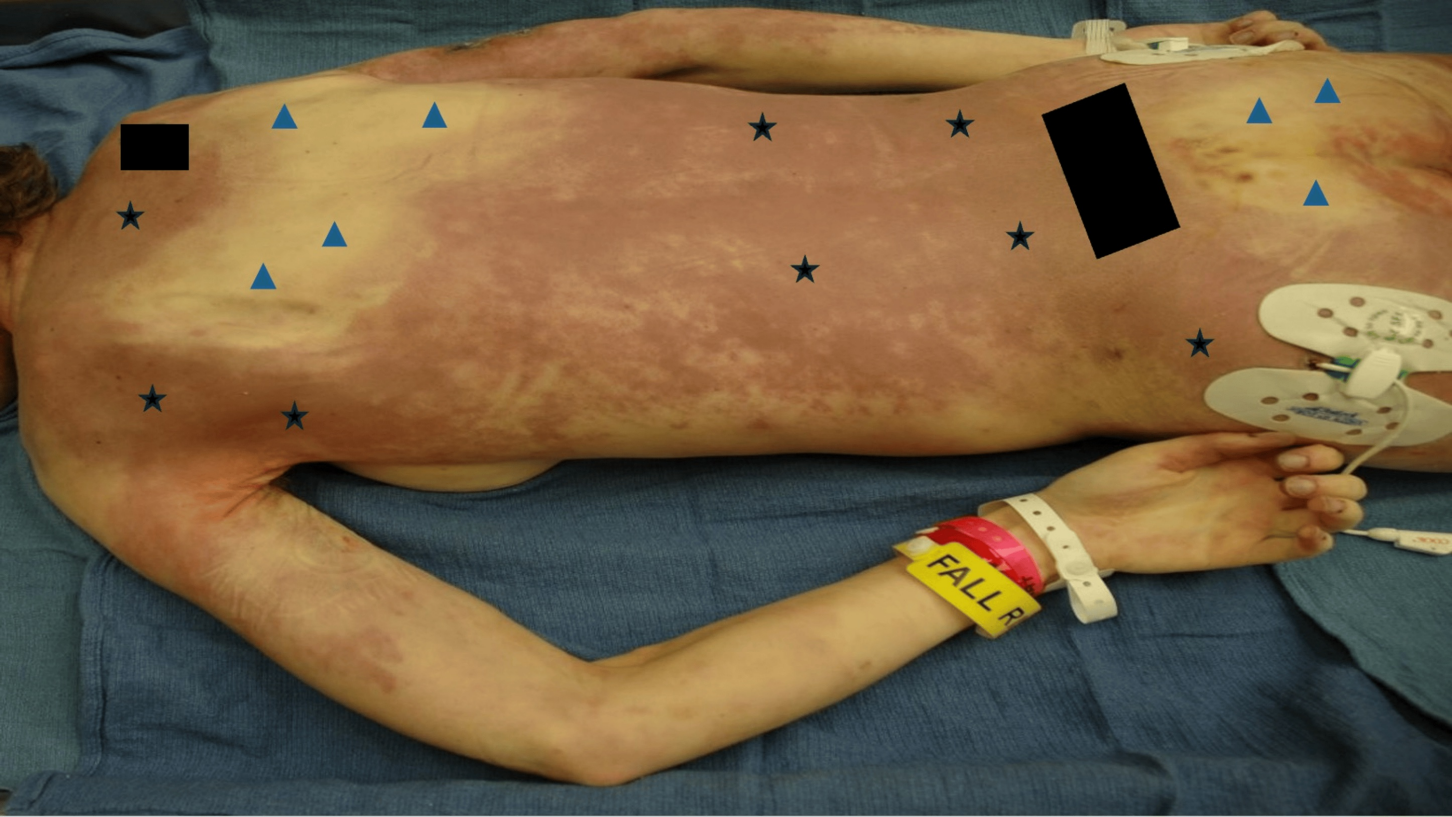
Livor mortis on the back and buttocks of a woman The lividity is red and fixed (black stars). The decedent was lying on her back and there is sparing of redness (contact pallor) in the areas that had pressure against them (blue triangles) that were located not only on her upper back (bilaterally overlying the scapular regions in a butterfly distribution) but also on her buttocks. The solid black rectangles are covering tattoos. Source: Author

**Figure 2:**
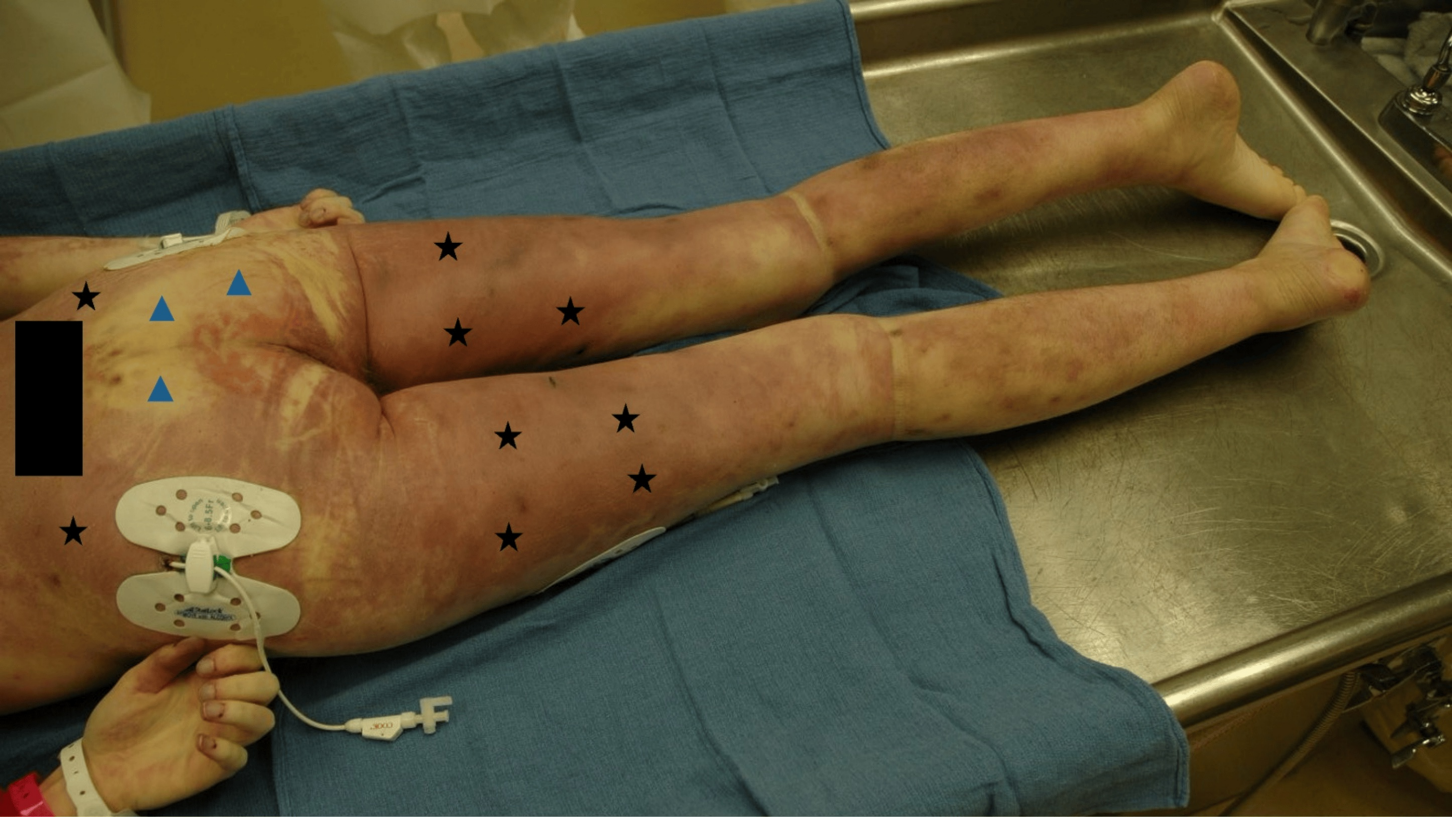
Postmortem hypostasis on the buttocks and legs of a female decedent The buttocks and posterior legs show fixed livor mortis that appears as diffuse, non-blanchable red lividity (black stars). The livor mortis spared the buttocks; the contact pallor (blue triangles) occurred in the buttock area since that location was exposed to pressure when she was lying on her back. The solid black rectangle is covering a tattoo. Source: Author

The red-purple discoloration of livor mortis is only present on the dependent areas of the body. Importantly, livor mortis does not occur on the skin located in areas that are under pressure. Therefore, the skin that is overlying an area that is pressing against a firm surface does not become discolored (Figures 1, 2).

Contact Pallor

**Figure 3:**
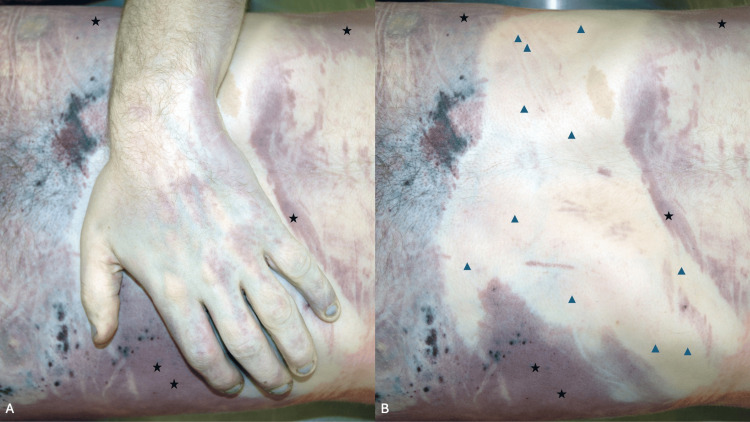
Contact pallor of the lower chest of a man A middle-aged man was lying face down. When he was found his livor mortis was fixed (black stars). He had been in a prone position; his left distal arm and hand were between his lower chest and the floor he was lying against (A). When he was turned over to a supine position, an absence of lividity (contact pallor) was observed on his lower chest (blue triangles) that corresponded to the location that his forearm and hand had covered which resulted in pressure that prevented the development of postmortem hypostasis (B). Source: Author

Contact pallor refers to the absence of lividity that results from continual pressure being applied to the skin. The absence of livor mortis in these areas is a permanent feature that remains unchanged (Figure 3). Hence, contact pallor can be useful for determining the position of the body after death [1-14].

The pattern of contact pallor of a supine deceased person lying with their face upwards, will typically demonstrate an absence of redness in a butterfly distribution on the upper back. Therefore, a decedent lying on their back would not demonstrate red discoloration of the skin overlying the scapula [1-14].

**Figure 4:**
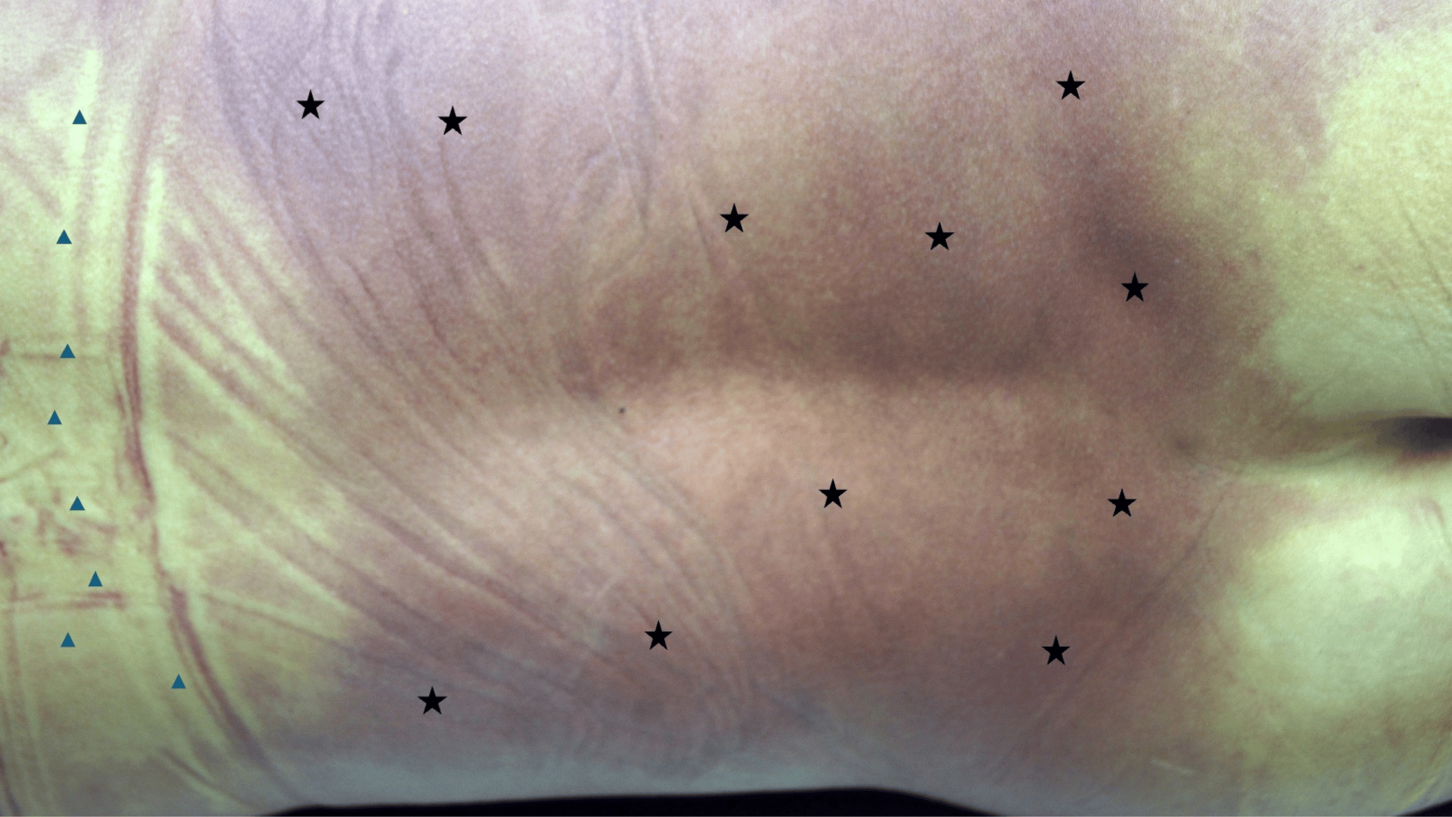
A young woman with confluent livor mortis on her lower back and contact pallor on the upper back Postmortem hypostasis presenting as diffuse redness from the mid back and extending to the lower back and proximal buttocks (black stars); the woman was lying on her back. She was wearing a bra which pressed firmly against her upper back; contact pallor (an absence of the lividity) developed in the areas of her back which were covered by the bra (blue triangles). Source: Author

Like a decedent lying on a firm surface, tight clothes that compress the skin and underlying soft tissues will prevent the development of livor mortis [1-14]. For example, a tightly fitting sports bra (Figure 4), or a very snug girdle, or compression stockings will prevent the occurrence of postmortem hypostasis to develop on the breasts, abdomen, or distal lower extremities, respectively [1-14]. In addition, livor mortis is diminished or absent in keloids or scars since these areas of tissue alteration have fewer vessels [1].

Shifting of Postmortem Lividity

**Figure 5:**
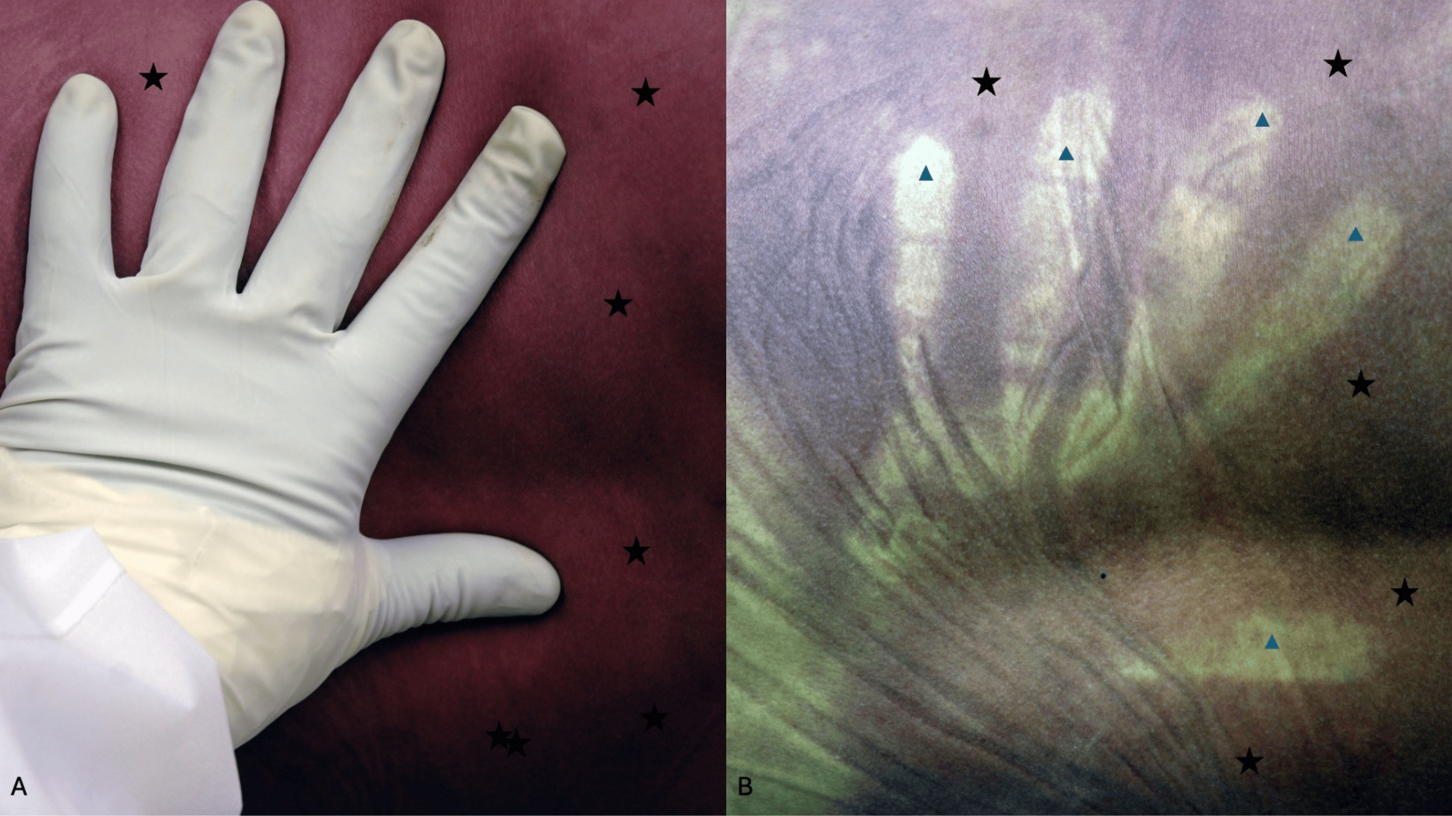
The blanching test to evaluate whether livor mortis is fixed The lower back and buttocks of a decedent who was found lying supine show confluent red lividity (black stars); the investigator used a gloved hand to press against the confluent red lividity (A). Livor mortis is not yet fixed since the area of confluent lividity demonstrates blanching of the redness (blue triangles) where pressure was applied (B). Source: Author

The status of rigor mortis can be evaluated by a blanching test. After postmortem hypostasis initially appears and during the time interval shortly after it becomes confluent, the red-purple lividity can be blanched by the pressure of a digit or hand being applied to the decedent’s skin (Figure 5). Once the digit or hand is removed, the redness will reappear [1-14].

Approximately eight hours after the time of death (and ranging from six to 12 hours), the result of the blanching test will be negative. The lividity will not be blanched after pressure from the investigator’s digit or hand is applied to the red skin of the decedent. The livor mortis that has developed is now permanent and will not be altered by changing the position of the decedent [1-13].

Before the blanch test becomes negative, the pattern of livor mortis can be altered. If the dead body is moved shortly after livor mortis appears or shortly after confluence of red-purple lividity has become established (for example within the first two to three hours), complete shifting of the lividity will occur, and the entire previous livor mortis will be relocated when prior postmortem hypostasis shifts downward to the new dependent areas. However, if the decedent is moved after confluent livor mortis has been present longer (for example after three to perhaps six hours) and the blanch test is still positive showing blanching of the red lividity after digit or hand pressure to the skin, incomplete shifting of the previously established postmortem hypostasis will occur; some of the initially observed lividity will remain in the former dependent area and new lividity will develop in the new dependent areas [1-14].

Fixed Lividity

Once the blanch test is negative, the observed livor mortis is referred to being fixed; changing the position of the body will neither alter the pattern of the lividity nor the presentation of the contact pallor. This occurs after approximately eight hours after death; however, it can range from six to 12 hours [1-13]. The fixed livor mortis can be useful in the death investigation of a decedent to determine whether the position of the body has changed after death has occurred or has been moved to another location [1-14].

Lividity becomes fixed because of two processes. It primarily occurs from the disintegration of the red blood cells in the blood vessels. Second, eventually hemoglobin diffuses into the perivascular tissue [3,10].

Sequelae of Livor Mortis

There are five stages of decomposition. Livor mortis is present in the first stage of decomposition (referred to as the fresh stage) which occurs from the time of death and lasts until approximately postmortem day two, with tremendous variability, especially based on environmental temperatures. The subsequent stages of decomposition include early decay (bloat) stage (days two to seven), active decay stage (days five to 13), advanced decay stage (days 10 to 23), and dry remains stage (days 18 to 90 and beyond) [3,7,9,10,12]. 

Postmortem hypostasis can become less obvious as decomposition of the body proceeds. Green discoloration of the skin occurs during the second (early decay) stage of decomposition. In addition, during this decomposition stage, the superficial veins demonstrate green marbling [3,7,9,10,12].

The green discoloration becomes dark brown/black during the third (active decay) stage of decomposition. Also in the third decomposition stage, there is slippage of the skin; the epidermis may be shed in a glove and sock pattern from the hands and feet, respectively. Once the decedent has entered the active stage of decomposition, livor mortis is usually less easily identified [3,7,9,10,12].

Unique clinical features of livor mortis

Tardieu Spots

**Figure 6:**
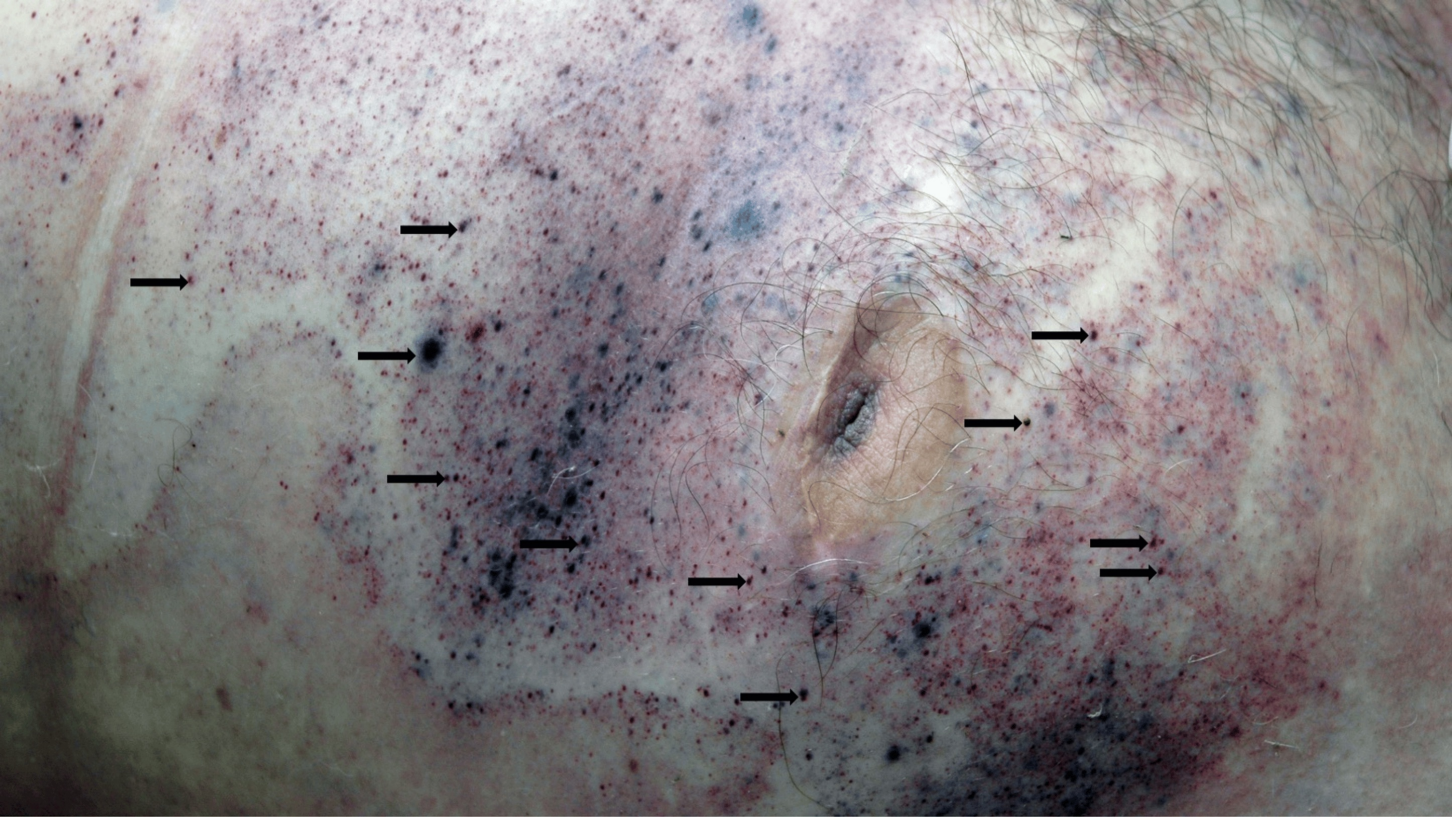
Tardieu spots on a man’s abdomen Purple petechiae and minute hemorrhages (black arrows) have developed on the periumbilical area the abdomen of a man who was lying prone after dying. Some of the smaller lesions have enlarged. The increased pressure of the blood on the blood vessel walls causes them to rupture. Source: Author

Auguste Ambroise Tardieu was a French physician and a forensic expert who had conducted more than 5000 autopsies. Tardieu spots describe the “petechiae” and minute hemorrhages that may occur in dependent areas, including those of livor mortis; the smaller lesions can enlarge and aggregate into patches of apparent purpura (Figure 6). Tardieu spots may be confused with petechiae and purpuric hemorrhages that occur premortem [1,6,7].

Tardieu spots commonly occur when the cause of death is hanging. They appear on the distal lower extremities of the fully suspended decedent. The mechanism of pathogenesis is that the small vessels rupture secondary to the increased pressure of the settling blood on the vessel wall [1,6,7].

Diminished Lividity

Livor mortis may not always be readily observed. Individuals who have severe anemia may not demonstrate postmortem hypostasis. Similarly, a person who dies because of extensive hemorrhage or who experience blood loss after death may not develop lividity [1].

Livor mortis may not be readily visualized in individuals who have a dark skin color [9]. Since livor mortis can be observed in fingernail beds, some forensic pathologists have suggested that the skin beneath the nail plate is a good location to evaluate a decedent who has a darker complexion for livor mortis [1]. The melanin present in the skin interferes with the visual detection of the red-purple lividity. Evaluation using a spectrophotometer may be helpful to document lividity and correlate the lividity with the time since death [8]. However, this technique is not yet available in most medicolegal death investigation offices.

Internal Livor Mortis

Lividity can affect the internal organs. This can usually be readily observed in the lungs; the dependent portion of the lungs will be deep blue to purple whereas the non-dependent areas of the lung will remain pink-tan. Aspirated blood will settle into the posterior aspects of the lungs if the decedent is lying supine. Pulmonary postmortem hypostasis can mimic pneumonia [4,12,13].

Similarly, in a supine decedent, the posterior heart areas of the heart will be deep purple due to congestion. When the hypostasis involves the left ventricle, this can be confused with a posterior myocardial infarction [4,8,12].

When the hypostasis affects the meninges, it can mimic a subarachnoid hemorrhage [12]. And, in the region of the occipital lobes and cerebellar hemispheres, engorgement of the vessels overlying the cerebral convexities can masquerade as an early stage of meningitis [4].

Hypostasis involving the intestine in a decedent who has died from hanging will show lividity in the most dependent loops of the small bowel [13]. Livor mortis of the intestines can mimic infarction, ischemia (such as tissue death caused by oxygen deficiency) or oxygen-related injury [8].

In a decedent who has died from hanging, the internal organs above the ligature (such as the larynx and tongue) may show a red-purple suffusion [8].

Lividity at the level of the larynx, behind the esophagus, may be misinterpreted as strangulation-related injuries. The dependent vascular congestion may result in foci of soft tissue hemorrhage. The hemorrhages can develop on the anterior surface of the cervical vertebrae and the posterior surface of the esophagus [4].

Intense hypostasis can develop on the skin of the dependent areas if the decedent dies with their head and neck in a dependent position. In this setting, the vessels of the scalp can be extremely congested. Therefore, the internal lividity can mimic a diffuse contusion on the surface and undersurface of the scalp [4].

Differential diagnosis of livor mortis

Bruises

The clinical differential diagnosis of livor mortis includes a bruise; indeed, under certain circumstances, dependent lividity may be confused with a contusion. Simply stated, the blood pools inside the vessels in postmortem hypostasis. In contrast, in a bruise, the blood is located outside the vessels; blunt force impact has resulted in the rupture of the blood vessels and the blood has exsanguinated into the surrounding tissues. Several characteristics can be used to differentiate livor mortis from a bruise (Table 2) [4,6,9,12].

**Table 2:** Differentiating livor mortis from a bruise ^a^The color of a bruise will vary depending on the age of the bruise. It originally may be pinkish red, then darker blue or purple or black, then violet, then green, then dark yellow, then pale yellow (or yellow-brown or light brown). ^b^In contrast to indistinct border of developing livor mortis, the border of an acute bruise is usually well-defined. ^c^The results of the blanching test prior to livor mortis becoming fixed. The blanching test is performed by pressing a digit firmly on the affected area and observing whether blanching occurs. ^d^The findings on gross examination (regarding the absence or presence of hemorrhage) into the cut soft tissue after an incision with a scalpel blade has been made into the affected area. Faint erythema, resulting from the congested vessels, may be observed after an incision into livor mortis; hemorrhage into the soft tissue is absent. ^e^The microscopic findings of a bruise depend on the age of the bruise. An early bruise will show extravasation of red blood cells into the dermis and subcutaneous fat. Inflammatory cells will eventually also be present in the dermis including polymorphonuclear leukocytes (neutrophils) and macrophages. Eventually hemosiderin-laden macrophages will be noted. Hematoidin granules may subsequently be observed.

Characteristic	Livor mortis	Bruise	References
Color^a^	Purple to red	Purple to blue	[4,6,9,12]
Location	Dependent parts	Anywhere, often in prominent parts	[4,6,9,12]
Border^b^	Indistinct	Well-defined	[4,6,9,12]
Local swelling	None, area flat	Present (accumulation of blood in area)	[4,6,9,12]
Depth in skin	Always superficial	Usually deep	[4,6,9,12]
Blanching test^c^	Area blanches	No blanching of area	[4,6,9,12]
Incision findings^d^	No hemorrhage; faint erythema may be present	Diffuse hemorrhage into soft tissue	[4,6,9,12]
Microscopic findings^e^	Erythrocytes in vessels	Erythrocytes in dermis, subcutaneous fat or both	[4,6,9,12]

The color of livor mortis is purple to red. In contrast, the color of a bruise will vary depending on the age of the bruise. The bruise originally may be pinkish red, then darker blue or purple or black, then violet, then green, then dark yellow, and then pale yellow (or yellow-brown or light brown) [4,6,9,12].

The depth of lividity and a bruise in the skin can vary. Postmortem hypostasis is always superficial. A bruise usually extends deeper into the skin of the affected area [5].

The border of livor mortis and that of a bruise are different. Livor mortis has an indistinct border. However, the border of an acute bruise is usually well-defined [4,6,9,12].

The presence of absence of swelling of the affected area is different in livor mortis and a bruise. There is no swelling in livor mortis; the area of lividity is flat. In contrast, swelling may be present in a bruise; it is caused by an accumulation of blood in the area [5].

The location of livor mortis and a bruise may differ. Lividity occurs in dependent parts of the body. A bruise can occur anywhere; more often it will be located in prominent parts of the body [5].

A clinical test of the affected area that can distinguish livor mortis from a bruise is the blanching test. The blanching test is performed by pressing a digit firmly on the affected area and observing whether blanching occurs. The results of the blanching test, prior to livor mortis becoming fixed, demonstrates blanching of the tested area in postmortem hypostasis. When pressure is applied to a bruise, the area does not blanch [4,6,9,12].

If lividity cannot be definitively differentiated from a bruise based upon appearance or a blanching test, an incision with a scalpel blade has been made into the affected area. The findings on gross examination of the cut tissue in livor mortis may show faint erythema; this results from vessels being congested; hemorrhage into the soft tissue is absent. However, when a bruise is incised, diffuse hemorrhage into the soft tissue is noted [4,6,9,12].

Occasionally, it may be necessary to evaluate the tissue microscopically. The microscopic findings of lividity are sparse, and the features may mimic those observed in normal skin; the vessels may be dilated, and numerous red blood cells are only noted to be present within the blood vessel. In contrast, the pathologic changes demonstrated by a bruise depend on the age of the lesion. An early bruise will show extravasation of erythrocytes into the dermis or subcutaneous fat or both. Inflammatory cells will eventually also be present in the dermis including polymorphonuclear leukocytes (neutrophils) and macrophages. Eventually hemosiderin-laden macrophages will be noted. Hematoidin granules may subsequently be observed [4,6,9,12].

Livor mortis is a feature of the first stage of postmortem decomposition; this stage is also referred to as the fresh stage (during postmortem days one to two). Once postmortem decomposition has progressed to the early decay (bloat) stage (days two to seven) and active decay stage (days five to 13), it may be difficult or impossible to differentiate livor mortis from a bruise. As the later stages of decomposition, there is diffusion of the hemolyzed blood from the blood vessels into the surrounding soft tissue; even microscopic examination of the affected areas will not be able to differentiate postmortem hypostasis from a contusion [4].

Body Coloration Artifacts

A review of the autopsy data from the archives of the Department of Forensic Medicine of All India Institute of Medical Sciences, New Delhi and Maulana Azad Medical College, New Delhi during a 12-year period (from 2004 to 2015), identified numerous cases in which misinterpretation was caused by external body coloration. Specifically, several decedents were identified with body coloration artifacts that mimicked postmortem livor mortis [22].

The source of the purple color occurred during the *Holi* color festivity. *Holi* is a color festivity of Hindus; it is celebrated in India and Nepal. *Holi* has now become an international festival and is celebrated in many of the countries in the Indian subcontinent. During the celebration, as a gesture of love and affection, people pour various colors over each other. Indeed, when the red-purple colors are used over the body a dead person, the appearance of the decedent can be confused with postmortem lividity [22].

Differentiating *Holi*-associated coloration artifact from postmortem changes was not always readily accomplished. Often a combination of evaluations was required. These included seeing he body before the washing of the color, a history of the application of the color, and various additional features including the usual appearance, distribution, and sites of the color [22].

The cases that were misinterpreted as livor mortis had purplish color diffusely over the shoulder, back and sometime in patches over the front of the lower limbs. In all decedents, the purple color had been used at the *Holi *color festival. The differentiating features that enabled the investigators to determine that the purplish color was the result of external body coloration were: (i) that the color faded out when scrubbed with normal water, (ii) the color represented an unusual lividity color for a tuberculosis patient, (iii) microscopic examination didn’t show blood stasis of capillaries and venules, and (iv) the worn clothes were also discolored [22].

Factors that affect livor mortis

The ambient temperature can affect the rate of the development of livor mortis. When the environmental temperature is warm, the appearance of lividity can occur sooner after the death and progression of hypostasis can proceed more rapidly. In contrast, when the decedent is in a location where the temperature is cooler, hypostasis may be delayed in appearance and lividity confluence or fixation or both may develop at a slower pace [1,6].

In addition to higher ambient temperature, several factors can accelerate the onset of livor mortis and the progression of lividity (Table 3) [1,6]. These include abnormal cardiac function (from pre-existing blood pooling due to cardiac problems), environmental processes (such as tight-fitting clothing and being in a confined space), larger blood volume, and body position (with livor mortis developing in dependent areas more rapidly) [1,6].

**Table 3:** Factors that can accelerate the onset of livor mortis ^a^Conditions associated with pre-existing blood pooling due to cardiac problems. ^b^This includes wearing tight-fitting clothing or being in a confined space. ^c^Livor mortis develops in dependent areas more rapidly.

Factor	References
Higher (warm) ambient temperature	[1,6]
Larger blood volume	[1,6]
Abnormal cardiac function^a^	[1,6]
Environmental processes^b^	[1,6]
Body position^c^	[1,6]

In addition to lower ambient temperature, several factors can decelerate the rate of livor mortis (Table 4) [1,6]. These include body position (if the body is not in a supine position), certain medical conditions (such as chronic illnesses including hypoxia and anemia, dehydration, and fever), limited blood supply (such as blood loss during death or after death), presence of clothing, and pressure on the body [1,6].

**Table 4:** Factors that can decelerate the onset of livor mortis ^a^These include chronic illnesses such as hypoxia and anemia, dehydration, and fever. ^b^This includes blood loss during death or after death. ^c^The rate of livor mortis is slowed when the body is not in a supine position.

Factor	References
Lower (cold) ambient temperature	[1,6]
Medical conditions^a^	[1,6]
Clothing	[1,6]
Limited blood supply^b^	[1,6]
Body position^c^	[1,6]
Pressure on the body	[1,6]

Forensic applications of livor mortis

There are three potential applications of livor mortis in forensic medicine. First, lividity can be used to estimate the time since death. Second livor mortis can be very helpful to establish the position of body when the individual died and whether the decedent has been moved after death. Third, the color of the lividity may possibly provide information regarding the cause of death [1-13].

Postmortem Interval Estimation

Observations related to livor mortis can be used when attempting to estimate the time since death; however, there is significant variability to the onset time of lividity. Also, the duration of time necessary for postmortem hypostasis to become fixed in any person is not uniform. Therefore, as a single modality, livor mortis cannot be used as a definitive method to establish the postmortem interval [4,15].

However, livor mortis may be useful in criminal investigations when the decedent is discovered after the appearance of lividity until postmortem hypostasis is fixed. The appearance of livor mortis can be as early as 20 minutes; however, it is typically able to be observed within two hours after death. Livor mortis may be fixed as early as six hours postmortem, but fixed lividity may not occur until 12 hours from the time of death [1-13].

In any individual the rate of onset of livor mortis is variable. Indeed, there are several factors that can either accelerate or decelerate the onset of lividity [1,6]. Therefore, finding a dead body between the appearance and fixation of livor mortis may allow the crime scene investigator to estimate the postmortem interval to be between approximately two hours and eight hours [1-13].

Position of Body at Death

Livor mortis can be very useful in establishing the position of the body at the time of death (or more specifically, the time at which lividity became fixed). Once lividity is fixed, the pattern of postmortem hypostasis can be used to determine if the body has repositioned. In addition, the presentation of livor mortis and contact pallor can be evaluated to establish whether the decedent has been moved from one site where the death occurred to another location [1-14,23].

If lividity is partially fixed, variable patterns of redness will be observed based on the initial dependent areas and then on the more recently established dependent locations. This has been referred to as incomplete shifting since some of the lividity remains in the former dependent areas and some of the hypostasis shifts to the areas that have newly become dependent [2]. In addition to the changes observed on the skin, variable patterns of postmortem hypostasis may be observed on the internal examination of the body’s organs [8].

Examples of livor mortis being used to elucidate the cause of death have been published. Dr. Frederic Thomas while presenting the Douglas Kerr Memorial Lecture in 1963 described the murder of a young lawyer, Bernays, that occurred in 1882 and had been included in the chapters on postmortem changes in most French and German textbooks [24]. Dr. Francis E. Camps in 1959, an English pathologist, described the case of Emmett Dunne who murdered Sergeant Watters [25].

Bernays murder: Dr. Thomas, a professor of Legal Medicine at the University of Ghent, Belgium shared the case of the murder of Mr. Bernays that occurred in Brussels that had been staged to appear as a suicide. The murderer was Mr. Leon Pletzer; his brother (Mr. Armand Pletzer) was in love with the wife of Mr. Bernays. Mr. Leon Pletzer shot Mr. Bernays on January 7, 1882; the bullet severed the brain stem. The body was discovered 12 days later in an armchair in a sitting position with the pistol next to it so that the setting would be interpreted as a suicide. The distribution of the livor mortis was over the entire back of the decedent and not from below the waist as would have been expected if Mr. Bernays had died while sitting in the chair. The pathologists determined that the decedent had remained on his back (for at least two or three days) until the muscle stiffening of rigor mortis had resolved. Mr. Armand Pletzer had come to the scene of the crime a few days after the murder and had staged the scene to mimic a suicide by placing Mr. Bernays in the chair [24].

Watters murder: Dr. Camps had several professional accolades including professor of Forensic Medicine at the London Hospital Medical College; in addition, he had authored several articles on forensic in the book Practical Forensic Medicine. A year after the death of British Sergeant Watters, he was able to demonstrate that the apparent suicide was a murder. Watters had a German wife (Mia) who reported that Watters had failed to return home on the night of November 30, 1953; in the early hours of the following morning, a search (by Sargeant Fry accompanied by Command Sargeant Major Emmett Dunne) discovered Watters hanging from the banister of a staircase in a building on the army base in Duisburg. The original autopsy gave the cause of death as “Asphyxia and /or Shock due to Hanging” and the manner of death recorded was “Suicide [25].”

In June of 1954, Dunne married Watter’s widow (Mia); this prompted an investigation into the death of Watters. The investigating officer asked Dr. Camps if he could differentiate whether the hanging of Watters occurred before or after his death. Examination of the photographs that had been taken “showed quite clearly that there was hypostasis and congestion both above and below the ligature mark quite inconsistent with a man who had been suspended for some hours [25].” Additional evaluation after exhumation demonstrated that the vertical fracture of the thyroid cartilage could not have occurred from strangulation and had resulted from a blow across the front of the neck that was performed with a linear object [25].

Dr. Camp’s conclusions included: “(i) the man had not died of hanging but had been suspended after death, and (ii) shortly after death the body had been in a position in which the head, neck and upper part of the chest had become extremely congested, probably by gravity [25].” After this information had been received, Dunne confessed to killing Watters and leaving the body at the scene. Additional information was secured that a second person assisted in holding up the body while Dunne used a rope to hang it from the banister above. The Government of Great Brittain convicted Dunne of murder and sentenced him to death; however, the Federal Republic of Germany commuted the sentence to life imprisonment [25].

Cause of Death

When the manner of death is natural, livor mortis is often red-purple; eventually the color may become deeper purple after lividity becomes fixed [1-14]. In certain circumstances when the manner of death is accidental, homicide or suicide, the skin discoloration of livor mortis may be different (Table 5) [1-14, 26-43]. Indeed, the skin color may provide salient information regarding the cause of death, such as carbon monoxide inhalation, hypothermia or ingestion of poison [1,5,12,26-43].

**Table 5:** Factors associated with color of livor mortis ^a^Mortuary refrigeration can result in the development of cherry-red lividity [1,5,12].

Cause of death	Color of lividity	Mechanism	References
Aniline	Deep blue	Methemoglobin production	[5]
Carbon monoxide	Cherry-red to pink	Carboxyhemoglobin production	[5,12,26-32]
Cyanide	Cherry-red to pink	Oxygen dissociation from hemoglobin inhibition by blocking cytochrome oxidase	[12,26,33,34]
Fluoroacetate	Cherry-red to pink	Inhibition of oxidative cellular metabolism	[1,35]
Hydrogen sulfide	Green	Sulfhemoglobin production	[1]
Hypothermia^a^	Cherry-red to pink	Oxygen dissociation from hemoglobin inhibition	[12,26,33,36-42]
Natural	Red to purple	Normal hemoglobin in the blood.	[1-14]
Phosphorus	Yellow-brown	It affects ribosome function, leading to defective protein synthesis	[12]
Potassium chromate	Brown	Generate reactive oxygen species that damage cellular components	[5]
Sodium chlorate	Brown, slate-gray	Methemoglobin production	[1,39,40]
Sodium nitrite	Brown, gray, purple, red brown	Methemoglobin production	[5,41-43]

Cherry-red to pink lividity may be observed in individuals who died from carbon monoxide intoxication. A similar cherry-red to pink appearance of postmortem hypostasis can result from hypothermia or refrigeration or immersion in water [1]. Poisons, such as cyanide and fluoroacetate, have also been noted to cause cherry-red to pink livor mortis [1,5,12,26-32,35]. Livor mortis can present with other skin colors such as brown (sodium nitrite, potassium chlorate, or sodium chlorate), deep blue (aniline), green (hydrogen sulfide which results in sulfhemoglobin production), red-brown (sodium nitrite), slate gray (sodium chlorate), or yellow-brown (phosphorus) [1,5,12,39-43].

Carbon monoxide: Carbon monoxide is a nonirritating gas; it is also colorless and odorless [5,12,26-32]. It is inhaled into the body [29]. Some of the sources of exposure to carbon monoxide include automobile exhaust, gas heaters, gas-powered equipment (such as air compressors), and house fires [29].

In the human body, the primary binding of carbon monoxide is to the iron molecules in hemoglobin to form carboxyhemoglobin. The affinity of carbon monoxide for hemoglobin is between 200 and 250 times greater than that of oxygen. In addition to binding to the iron molecules in hemoglobin, carbon monoxide also binds to the iron in myoglobin and cytochrome C [29].

When the level of carboxyhemoglobin is greater than 50 percent, severe or life-threatening symptoms may be associated with the carbon monoxide toxicity [29]. A distinctive cherry-red to pink discoloration of skin can be observed in individuals who die from carbon monoxide intoxication [5,26-28]. In addition to water immersion and strangulation, postmortem pink teeth may be observed in decedents who die from carbon monoxide intoxication [30].

In a study of 182 unintentional carbon monoxide-related deaths, 98 percent (179 decedents) demonstrated cherry-pink lividity. The investigators noted that a clear cherry-pink coloring of livor mortis was observed in the fresh corpses with carboxyhemoglobin levels greater than 31 percent. The researchers concluded that, based on the color of the lividity, coroners at the death scene, should immediately be able to recognize carbon monoxide-related deaths [28].

In another study of only 10 fatal cases of carbon monoxide poisoning, the researcher only observed cherry-red lividity in five of the decedents. Several reasons were postulated for the inability to detect the cherry-red postmortem hypostasis. The possible causes included the skin pigmentation of the decedent, that an impression of cyanosis was produced by deep venous dilatation with superficial vasoconstriction, a low carbon monoxide concentration, or washing out of a previously high concentration of carbon monoxide [27].

Cyanide: Cyanide (hydrogen cyanide) can be absorbed through oral, inhalational, dermal, or parenteral routes. Cyanide is a potent decoupler of oxidative phosphorylation; poisoning causes the disruption of adenosine triphosphate (ATP) synthesis by interfering with the action of cytochrome C oxidase. Oxygen dissociation from hemoglobin results and there is subsequent inhibition of aerobic respiration [12,26,33,34].

In a retrospective review of 65 articles (which included 102 patients), cutaneous manifestations of cyanide poisoning were not commonly observed. Cherry-red or pink skin was only described in 11 percent of the decedents. Cyanosis was only noted in 15 percent of the cases (15 individuals) [34].

A distinctive odor may be associated with cyanide poisoning [26,34]. This was observed in only 15 percent of cases. Seven individuals described a bitter almond odor on the breath. Other terms used to describe the odor included burned, unpleasant, ammonia-like, musty, and putrid. Importantly, many individuals cannot discern the odor; previous studies of odor detectors has ranged from 20 to 40 percent or possibly 60 to 80 percent [34].

Fluoroacetate: Fluoroacetate has been used as an insecticide and a rodenticide. Its mechanism of action is by inhibition of oxidative cellular metabolism. Once in the human, it is converted to fluorocitrate. This extremely toxic compound is a competitive inhibitor of the enzyme aconitase in the tricarboxylic (Krebs or citric) acid cycle. A pair of investigators have listed cherry-red to pink lividity to be associated with fluoroacetate poisoning [1,35].

Hydrogen sulfide: Two researchers listed green lividity to be associated with hydrogen sulfide poisoning. The production of sulfhemaglobin resulted in the green discoloration [1].

Hypothermia: Hypothermia is defined when the core body temperature is below 95 degrees Fahrenheit (or 35 degrees Centigrade). Importantly, at autopsy, there are no pathognomonic signs of fatal hypothermia. Some autopsy findings that may be associated with hypothermia include cherry-red to pink livor mortis (resulting from the inhibition of oxygen dissociation from hemoglobin), frost erythema on gross examination (red apparent injuries of the extensor side of large joints, such as the elbows and knees), and Wischnewski spots (also known as leopard spots) which are hemorrhagic spots of the gastric mucosa [12,26,33,36-42].

A coroner provided an excellent description of hypothermia-related livor mortis. “In persons dying in a cold environment (the alcoholic who goes to sleep in a snowbank is the typical example), the skin and dependent lividity may have a bright scarlet hue, simulating the cherry-red color of carboxyhemoglobin. Other findings in acute cases include nonspecific changes associated with asphyxia-pulmonary congestion and edema, froth in the air passages and subendocardial petechiae. There are no pathognomonic changes [26].”

Other researchers provided a description of the livor mortis color they observed in a man who died from hypothermia. The minimum temperature overnight was estimated at 4.0 degrees Centigrade. A man in his early 60’s was found to be adequately dressed in a jacket and pants; however, his hat and shoes were located nearby. The “pinkness of hypostasis” was observed on external examination [36].

Frost erythema (also referred to as cold erythema) presents as reddish purple to violet or brownish brown skin discoloration that are mostly located over the extensor surfaces of the large joints such as those of the elbows, knees, and greater trochanter of the femur region of the hip. It can also be observed on the face localized to the sites that project such as the ears, nose and zygomatic area. Rarely, frost erythema occurs on the male external genitalia [38].

Refrigeration: Like hypothermia, cherry-red or pink lividity can result from refrigeration [1,5,12]. A group of investigators stated that “refrigeration may also produce pink hypostasis [5].” A pair of researchers commented that cherry-red livor mortis can be an “artifact of mortuary refrigeration [12].” Another pair of investigators also list cherry-red or pink lividity to be associated with refrigeration and attribute the oxygen retention to result from the cold air [1]. Thus, in certain instances, bright red lividity is merely a postmortem event, and can occur in any death, whether non-natural or natural, so long as refrigeration occurs soon after death.

Sodium chlorate: Sodium chlorate produces methemoglobinemia. It was previously used as an insecticide, a raticide, and a herbicide. The associated manner of death from sodium chlorate included suicide and homicide [1,39,40]. Cyanosis typically accompanied livor mortis. The murder victim, a 64-year-old man, not only had patchy cyanosis on his face, but also a dusky brown lividity [40]. Two researchers also listed brown lividity to be associated with sodium chlorate [1].

Two investigators reported three cases of suicidal poisoning by sodium chlorate. The men were age 19 years, 43 years, and 57 years. Each man had marked and prominent cyanosis. Each man also had slate gray lividity [39].

Sodium nitrite: Like sodium chlorate, sodium nitrite (NaNO2) and sodium nitrate (NaNO3) are both inorganic compounds that can cause acquired methemoglobinemia; sodium nitrite is used as a preservative in the fish and meat industry. The compound oxidizes the iron component of hemoglobin, forming methemoglobin, methemoglobinemia, and tissue hypoxia. Other drugs that can result in methemoglobinemia include benzocaine, lidocaine, sulfonamides, paraquat, dapsone, chlorate and aniline [5,41-43].

The normal level of methemoglobinemia is less than three percent. Between three to 10 percent, the skin color becomes pale gray to blue. From 10 to 30 percent, there is cyanosis with dark discoloration of the blood and confusion. When the levels greater than 70 percent, there is severe hypoxemia and death [42].

 A group of investigators described an 18-year-old woman who died of suicide after ingesting sodium nitrite. External examination of the lips and fingernails revealed cyanotic changes. On the back of her body was gray-brownish livor mortis [42].

A retrospective study of 28 deaths from either sodium nitrite or sodium nitrate ingestion was particularly remarkable for the characteristic gray-purple lividity. The lividity coloring included variations of purple, gray, and sometimes brown; the lividity was unremarkable in seven cases. Purple-gray, gray and purple-brown lividity were observed in five, three, and two cases, respectfully. The postmortem hypostasis of individual decedents was either grayish brown, red-purple-brown, dark purple-blue, dusky gray, purple-brown-gray, gray blue-purple, maroon-brown, gray-green-blue, ash-colored, and blue-gray [41].

New advances regarding livor mortis: spectrophotometric analysis of postmortem lividity

Currently, the blanching test by applying pressure using a digit/hand of the investigator is used to assess lividity [1-13]. Since this method of assessment is subject to subjective variability, it can be difficult to use livor mortis to estimate a range of time since death for a decedent [1-13]. New advances regarding the evaluation of lividity (that incorporates the assessment of hemoglobin in the area of hypostasis) and the use of spectrophotometry to correlate the objective determination of lividity and the postmortem interval has significantly improved the possibility of more accurately establishing the estimated time since death using livor mortis [23,44-47].

The concept of assessing hypostasis by colorimetry was introduced by Vanezis in 1991; subsequently, in 1996, Vanezis and Trujillo provided additional evidence of the usefulness of a colorimeter measuring system could be used in the assessment of the postmortem interval [23]. They used a tristimulus colorimeter. They found that the change of lightness decreased as the postmortem period increased and the shift in hypostasis was most marked in the first 12 hours since death [23].

Usumoto et al. proposed an equation to be used to estimate the postmortem interval based on the spectrophotometric analysis of postmortem lividity in 2010 [44]. Their equation provided an accuracy of estimating the time since death within 4.76 hours. The calculations were based on the L*a*b* color system introduced in 1976 by the Commission Internationale de l’Eclairage [44].

In a follow-up study published in 2019, Usumoto examined how postmortem lividity was influenced by blood color. They used equations (with and without blood color values) to estimate the color of postmortem lividity and the postmortem interval. They found that L* (which represents Value) of postmortem lividity and control skin were strongly correlated. They also found that a* and b*(which represent Chroma and Hue) of postmortem lividity significantly increased with an increasing postmortem interval; however, each of the results was associated with a low coefficient of determination (R2 value). In conclusion, the investigators suggested that to achieve a better understanding of the mechanisms of postmortem lividity, further study was necessary to understand the factors that influence postmortem lividity [45].

More recently, in 2022, De Donno et al. used spectrophotometry to assess the intensity of hypostasis and discoloration (which they refer to as paleening) in Caucasian cadavers. The investigators used a dynamometer to apply a standardized force to the skin surface of the cadaver and a spectrophotometer (Antera 3D) to evaluate only the hemoglobin in skin color (while simultaneously excluding melanin). They found a statistically significant percentage reduction in hemoglobin (red% Hb, determined by comparing the hemoglobin after weight force application to the hemoglobin before weight force application) in the skin areas analyzed and the time since death. Based on their results, they developed a mathematical formula to estimate the time since death (Y) by incorporating the degree of paleening of the hypostasis. The formula had a reliability range of 4 hours and 50 minutes; the formula was: Y = (-23.034 x red% Hb) + 13.676 [46].

Calvano, Introna, and De Donno commented that postmortem lividity cannot be accurately estimated in individuals whose skin has a darker color. In 2024, they incorporated the methodology that De Donno et al. had previously developed. Their intention was to determine whether their original observations could be applied to decedents with skin of color [47].

They evaluated a 21-year-old man who had been born in Ghana with a Fitzpatrick skin phenotype of V-VI; the decedent had drowned. They used the same Antera 3D spectrophotometer (which could exclude the contribution of the melanin pigment to the skin color). They observed that their spectrophotometry protocol that they had used in the Caucasian decedents could be used for the evaluation of livor mortis to estimate the time since death for individuals in the negroid ethnic race [47].

Thus, despite the ongoing progress being made with regard to spectroscopic investigation of lividity in order to determine more accurate estimates of the time of death, with results that have accuracies on the order of +/- greater than four hours, the practical utility of such techniques is questionable at this time.

## Conclusions

Livor mortis is a postmortem change. The onset can be noted as early as 20 minutes after death; however, it is usually observed within two hours after the person has died. After four to six hours, it is readily apparent, and after eight to 12 hours, it is usually fixed. The onset and duration until fixation of postmortem hypostasis are variable; lividity is also influenced not only by temperature but also by other factors. Therefore, as an independent observation, livor mortis is not reliable for establishing the estimation of a range of time since death; however, in combination with information from other postmortem changes, such as rigor mortis, it may be helpful in establishing a range for the postmortem interval. Livor mortis was initially described during the thirteenth century by the Chinese physician Song Ci in his book The Washing Away of Wrongs. Information regarding the onset and pattern of livor mortis has repeatedly been crucial in criminal investigations. A bruise may mimic some of the features of livor mortis; however, clinical features (such as blanching of postmortem hypostasis) can often differentiate the conditions. The color of livor mortis may be useful in determining the cause of death; cherry-red or pink lividity may be associated with carbon monoxide intoxication, hypothermia, or cyanide poisoning. Analysis of lividity incorporating the use of spectrophotometry is an important new advance regarding the use of readily available technology to provide objective measures to assess postmortem hypostasis that can be correlated to determining an accurate estimate for the postmortem interval; however, in its current state, application of this new technology is not yet practicable in most death investigation offices.
